# The impact of arbuscular mycorrhizal colonization on flooding response of *Medicago truncatula*

**DOI:** 10.3389/fpls.2024.1512350

**Published:** 2025-01-08

**Authors:** Vajiheh Safavi-Rizi, Helen Friedlein, Sayedhamid Safavi-Rizi, Franziska Krajinski-Barth

**Affiliations:** ^1^ Department of General and Applied Botany, Institute of Biology, Leipzig University, Leipzig, Germany; ^2^ Department of Plant Physiology, Institute of Biology, Leipzig University, Leipzig, Germany; ^3^ Department of Information Technology Engineering, Institute of Information Technology and Computer Engineering, University of Payame Noor, Isfahan, Iran

**Keywords:** flooding, arbuscular mycorrhizal symbiosis, climate change, *Medicago truncatula*, phosphorus flooding tolerance, *Mycorrhizal symbiosis*, *Rhizophagus irregularis*

## Abstract

Climate change is expected to lead to an increase in precipitation and flooding. Consequently, plants that are adapted to dry conditions have to adjust to frequent flooding periods. In this study, we investigate the flooding response of *Medicago truncatula*, a Mediterranean plant adapted to warm and dry conditions. Arbuscular mycorrhizal (AM) symbiosis plays a key role in enhancing plant growth and stress tolerance, yet its interactions with environmental stressors such as flooding remain poorly understood. In this study, we investigated the effects of mycorrhizal colonization and flooding stress on the growth, physiological and molecular responses of *M. truncatula* wild-type (WT) and *ha1-2* mutant lines. *ha1-2* mutant plants are unable to form a functional symbiosis with AM fungi as they are impaired in the proton pump required for phosphate transport from AM fungus to the plant. Over a six-week period, WT and *ha1-2* plants were cultivated in the presence of *Rhizophagus irregularis* and subsequently subjected to a 10-day waterlogging treatment. Our results indicated that under control and also flooding conditions, WT mycorrhizal plants exhibited increased dry biomass compared to non-mycorrhizal WT plants. In contrast, the *ha1-2* mutant plants did not show the enhanced biomass gain associated with AM symbiosis. The decline in biomass in response to flooding was more pronounced in mycorrhizal plants compared to the non-mycorrhizal plants. In mycorrhizal plants, flooding suppressed the transcript levels of *MtPt4* gene in both WT, although not significantly, and *ha1-2* mutant lines. Gene expression analysis showed modulation in genes related to nitrogen metabolism and hypoxic response. A strong upregulation of the *MtGNS1* transcript (~3000-fold) was observed in WT, however, this upregulation was milder in the *ha1-2* plants. Our findings suggest that while AMF symbiosis positively affects plant biomass under control conditions, its beneficial effects were attenuated under flooding stress. Future research will focus on understanding the molecular mechanisms behind AMF modulation of flooding stress responses, including nutrient uptake and metabolism, stress tolerance, and recovery post-flooding. These results will facilitate the enhancement of AMF-based strategies to improve plant resilience against climate change-induced flooding events.

## Introduction

Flooding-induced hypoxia, oxygen deprivation, in plants poses a critical challenge to agricultural yields (Bailey-Serres et al., 2012). Intense precipitation and flooding primarily stem from drastic shifts in weather patterns, changes in land cover, and the elevation of sea levels (Roy et al., 2023). Extensive flooding induces a pronounced decline in vital physiological processes within plants, including stomatal conductance, photosynthesis, nitrogen fixation, shoot growth, root development, and nutrient absorption. This multifaceted impairment ultimately leads to substantial losses in plant yield (Kaur et al., 2020). At the cellular level, hypoxia disrupts mitochondrial redox balance and ATP generation and therefore hypoxic tissue adaptation includes internal organic electron acceptors, such as ethanol and lactate fermentation, to restore redox equilibrium and support ATP regeneration (Weits, 2021). In *Arabidopsis thaliana*, the oxygen sensing and signaling process is facilitated through the oxygen- and NO-dependent N-degron pathway, orchestrating the proteasomal degradation of subgroup VII ethylene response factors (ERFVII) during aerobic conditions. Stabilized in hypoxic environments, ERFVII transcription factors migrate from the cytosol to the nucleus, inducing the expression of hypoxia response genes, among them glycolysis and fermentation-related genes encoding alcohol dehydrogenase (ADH) and pyruvate decarboxylase (PDC) enzymes (Gibbs et al., 2011; Licausi et al., 2011). Ethylene enhances ERFVII stability by reducing NO levels leading to pre-adaption of plants to hypoxia (Hartman et al., 2019).

The symbiosis between plants and arbuscular mycorrhizal fungi (AMF), such as *Rhizophagus irregularis*, is shown to enhance plant growth and stress resilience including flooding (Fougnies et al., 2007; Krajinski et al., 2014; Wang et al., 2021). Soil inorganic phosphate (Pi) levels influence AM fungal colonization of roots. In low-P soils, AMF symbiosis becomes vital for supplying the majority of the required Pi for plants (Krajinski et al., 2014). Efficient Pi transport from fungus to plant cells relies on a proton gradient across the periarbuscular membrane (PAM) (Krajinski et al., 2002). Mutation of H^+^-ATPase gene, *HA1*, in arbuscule-containing root cells of *Medicago truncatula* (*ha1-2* mutant) led to impaired arbuscule development while retaining regular root colonization by *Rhizophagus irregularis* hyphae with no growth enhancement in response to mycorrhizal colonization under Pi limitation (Krajinski et al., 2014; Wang et al., 2014). Moreover, the uptake of nitrogen (N) from organic sources could play a crucial role in AMF symbiosis, benefiting both plant and fungal partners (Leigh et al., 2009). Studies in natural ecosystems have shown that AM fungal communities shift with inorganic N application, indicating that AM fungi are sensitive to soil N availability and play a role in N uptake, especially when there is competition for N (Egerton-Warburton and Allen, 2000; Leigh et al., 2009).

Gaining insights into how various organisms, including crop plants, respond to soil hypoxia during flooding and their interaction with symbiotic and widespread AM fungi is crucial for advancing plant yield and fostering sustainable agriculture (Maček, 2017). Flooding significantly influences the modulation of plant responses to AM fungi symbiosis by exerting effects on root architecture, soil conditions as well as the photosynthetic process, thereby impacting carbon levels in the host plant (Wang et al., 2021). There are few studies on the effect of AM symbiosis on plant flooding response with majority of them being conducted on rice, which shows high tolerance to flooding due to the formation of aerenchyma tissue (Solaiman and Hirata, 1995; Paszkowski et al., 2002; Vallino et al., 2014; Wang et al., 2021). It has been shown that AM colonization can have a positive effect on plant biomass, yield and P uptake under flooding (Zheng et al., 2020). Mycorrhizal Australian pine, *Casuarina equisetifolia*, seedlings adapted better to flooding than non-inoculated ones, showing increased adventitious root development and hypertrophied lenticels, which enhanced oxygen availability (Osundina, 1997). Mycorrhizal infection also reduced the accumulation of toxic byproducts like ethanol from anaerobic respiration (Osundina, 1997). However, negative effects have also been demonstrated (Wang et al., 2021). Flooding can inhibit the initiation of AM colonization in wetland grass roots, potentially through direct effects like reduced spore germination or fungal hyphal growth, or indirect effects such as increased root length, leading to lower carbon allocation to the fungi. However, once established, AM colonization seems unaffected by flooding over several months. This inconsistency requires a closer look at the interaction between plant and AMF under flooding conditions (Miller and Sharitz, 2000).

*Medicago* species serve as an important model legume and are able to form symbioses with nitrogen-fixing bacteria, rhizobia and AMF and is sensitive to flooding (Striker and Colmer, 2016). Here, we investigated the flooding response of *Medicago truncatula*, a perennial legume in Mediterranean climates (Inostroza et al., 2021).

We studied to what extent colonization with the AM fungi *Rhizophagus irregularis* affects the flooding tolerance of *M. truncatula*. The AMF *R. irregularis* is considered as an ubiquitous, flexible fungus, which also occurs in aquatic systems (Maček, 2017).

In the current study, we investigated the role of *R. irregularis* in modulating the *M. truncatula’s* response to hypoxic conditions, a common consequence of flooding. Moreover, through the use of the *ha1-2* mutant, which is unable to transport Pi to plant roots (Krajinski et al., 2014), we explored the impact of phosphate transport on the influence of *R. irregularis* symbiosis on Medicago’s response to flooding. Our physiological and molecular analysis provides insights into the regulatory networks involved in plant adaptation to flooding stress with and without *R. irregularis* symbiosis. These data lay the foundation for future studies aimed at harnessing the benefits of AM fungi symbiosis to improve crop resilience in the face of climate-induced challenges, particularly flooding.

## Materials and methods

### Plant material, growth condition and inoculation with *R. irregularis*

We utilized the Tnt1 insertion mutant, which is homozygous for *ha1-2* in the R108 ecotype (Krajinski et al., 2014). We compared it to the homozygous progeny lacking the *ha1-2* allele derived from the same parent, serving as wild-type controls (WT Tnt1) (Krajinski et al., 2014). *M. truncatula* WT and *ha1-2* seeds were germinated on 2% water agar with pH 7, following one night of storage at 4°C (Branscheid et al., 2010). The AM fungi inoculum was prepared by growing *Allium schoenoprasum* with *R. irregularis* following the method described previously (Krajinski et al., 2014). Inoculation with *R. irregularis* included blending the inoculum with a mixture of vermiculite and sterilized expanded clay in a ratio of 2:1. In the case of non-mycorrhizal treatments, an equal amount of vermiculite-clay substrate was employed, with the exclusion of the AM fungus. Nine Seedlings per treatment were transplanted in 11 × 11 × 12 cm black plastic pots filled with the above-mentioned substrate mixture. Three seedlings were grown in each pot and were pooled as one biological replicate. Plants were grown in a growth room in the biology institute of Leipzig University, located at coordinates 51.3311°N, 12.3939°E, with 150 μmol photons.m-2.s-1 and 25°C under a 16/8-h light/dark regime. Every other day, all plants received an equal amount of half-strength Hoagland nutrient solution containing 2.5 mM Ca(NO_3_)_2_, 2.5 mM KNO_3_, 1 mM MgSO_4_, 20 µM KH_2_PO_4_, 50 µM NaFeEDTA, 0.2 µM Na_2_MoO_4_·2H_2_O, 10 µM H_3_BO_3_, 0.2 µM NiSO_4_, 1 µM ZnSO_4_, 2 µM MnCl_2_, 0.5 µM CuSO_4_, and 0.2 µM CoCl_2_ (Hoagland, 1950). As defined by Branscheid and colleagues (Branscheid et al., 2010), 20 µM Pi is considered optimal for promoting arbuscular mycorrhizal fungal (AMF) colonization under the given culture conditions (Medicago, sand, clay, no soil). Additionally, a Pi concentration of 1/2 mM is still classified as mild Pi starvation. This characterization has been consistently established in various studies focusing on Medicago and its symbiotic relationships under these specific growth conditions, including works by Branscheid et al., 2010 (Branscheid et al., 2010) and Krajinski et al., 2014 (Krajinski et al., 2014).

### Waterlogging treatment

WT and *ha1-2* mutant lines were cultivated for six weeks in the presence of *R. irregularis* fungi. Following this initial growth period, the plants were subjected to a 10-day waterlogging treatment, during which the half-strength Hoagland nutrient solution, as described previously, was applied to cover the soil surface up to 1 cm above it. This treatment was sustained until the harvest day.

### Measurement of leaf Pi content

Pi determination was conducted according to Huang and Zhang, 2006 (Huang and Zhang, 2006) with minor modifications. In brief, frozen leaf materials were ground with liquid nitrogen. Plant extracts were prepared by adding 200 µl of 0.2 M NaOH to 20 mg of each sample, followed by vortexing for 5 min. The samples were heated at 96°C for 30 seconds and 200 µl of 0.25 M HCl was added. Subsequently, 100 µl of 0.5 M Tris-HCl pH 8 in 0.25% Tween 20 was added and heated at 96°C for 2 minutes. After centrifugation, the liquid above the plant material was transferred to new Eppendorf tubes and stored at 4°C. For color reagent preparation, calibration curve tubes received 225 µl of distilled water, 1500 µl 1M HCl, and varying volumes of a 500 µM KH_2_PO_4_ stock solution. Sample tubes were prepared similarly, with 225 µl distilled water and 1500 µl 1M HCl. Each sample was measured in three dilution levels. Staining solution (3 ml of 4.2% (NH_4_)_6_Mo_7_O_24_ in 5 M HCL and 9 ml of 0.2% malachite green) was added to all tubes. After vortexing, tubes were incubated at 30°C for 45 min in the dark. Measurements at 630 nm were taken using a spectrophotometer immediately after preparation. Pi concentrations in samples were determined using a KH_2_PO_4_ calibration curve, with calculations based on the fresh weight. Measurements were done in triplicate across three independent biological replicates.

### Measurements of dry biomass and chlorophyll content

Leaves and roots were placed in coffee filter bags and subjected to drying in a Heraeus Function Line drying cabinet (Heraeus) at 78°C for a duration of 48 hours. The resulting dry weight was determined using an analytical balance (Sartorius BP 210 D). To assess the relative chlorophyll levels in fully developed leaves, a SPAD-502 chlorophyll meter (Konica Minolta, Japan) was used. Three readings were taken from similar positions on a leaf for each biological replicate, and their average was calculated to represent the SPAD value.

### Quantification of arbuscular mycorrhiza

Microscopic determination of root colonization by *R. irregularis* was conducted following trypan blue staining (Phillips and Hayman, 1970). The quantification of various fungal structures was carried out with a bright-field microscope (Axioskop, ZEISS) at 40X magnification (Trouvelot et al., 1986). In brief, we analyzed three sections (the two ends and the middle) of each root sample from 10 root pieces per slide, resulting in a total of 30 views per slide. Fungal structures were identified according to the method described by Trouvelot et al. (1986). We estimated the percentage of root fragments exhibiting fungal structures and classified them into intensity classes (1-5) and arbuscule frequency classes (A0-A3). Each root fragment was assigned an intensity class, which was recorded in a table. If no fungal structures were observed, a “0” was noted. Each root fragment was evaluated in the same manner across the three sections, yielding 30 entries per table. We then counted the number of fragments in each intensity and arbuscular abundance class and calculated mycorrhizal colonization parameters using the Trouvelot et al. (1986) method (Trouvelot et al., 1986). Quantitative molecular analysis of *R. irregularis* within the root system was conducted through qRT-PCR, focusing on the and glutamine synthetase (*RiGNS1*) gene and ribosomal RNA (RirRNA) as housekeeping gene (Branscheid et al., 2010).

### RNA isolation, cDNA synthesis, and real-time PCR

Total RNA was extracted from frozen leaves and roots using the NucleoSpin RNA Plant and Fungi kit (Macherey-Nagel, Germany) following the manufacturer’s instructions. The concentration and purity of RNA were assessed using a NanoDrop ND-1000 photospectrometer (Thermo Scientific, Germany). A DNase I treatment was performed on 500 ng of RNA. Complementary DNA (cDNA) was synthesized with the RevertAid H Minus First Strand kit (Thermo Scientific) using oligo-(dT)18 primers. Following elution in 20 μl RNase-free water, samples were diluted by adding 20 μl deionized water, resulting in a final volume of 40 μl. RT-qPCR reactions were carried out in a total volume of 5 μl, containing 2.5 μl Power SYBR Green Master Mix (Thermo Scientific), 0.5 μM forward primer, 0.5 μM reverse primer, and 0.5 μl of cDNA. The reference genes encoding elongation factor alpha (EF1-α) and glyceraldehyde-3-phosphate dehydrogenase (GAPDH) were used for normalization in the data analysis of transcript levels for *M. truncatula* genes. Additionally, the transcript level of *RirRNA* served as the reference for normalizing the *R. irregularis* marker gene. The thermal profile for all RT-qPCRs included an initial denaturation step at 95°C for 2 min, followed by 40 cycles of denaturation at 95°C for 3 s and annealing/extension at 60°C for 30 s. Data analysis was performed using the 2^−ΔΔCt^ method (Schmittgen and Livak, 2008). The sequences of all gene-specific primers are provided in **Supplementary Table 1**.

### Statistical analysis

Statistical analyses for significant differences were conducted through two-way ANOVA using GraphPad Prism software (v. 9.4.1), assessing the impacts of *R. irregularis* and flooding. Subsequently, a Tukey’s multiple comparison test was employed to compare all growth conditions. For pairwise comparisons, Student’s t-test was applied (* p ≤ 0.05, ** p ≤ 0.01, *** p ≤ 0.001).

## Results

### Effects of AM symbiosis and flooding on growth and physiological response of WT and *ha1-2* lines

To determine the optimum flooding duration, we conducted a time-series experiment on wild-type (WT) plants without mycorrhiza. Plants were subjected to waterlogging for 3, 5, and 7 days. Phenotypic observations revealed that 7 days of waterlogging had a noticeable impact on plant phenotype, though it was not lethal (**Supplementary Figure S1**). Based on these observations, we selected a 10-day flooding period for subsequent experiments. This duration is slightly longer than 7 days, ensuring a significant effect of flooding while still allowing for plant survival.

**Figure 1:**
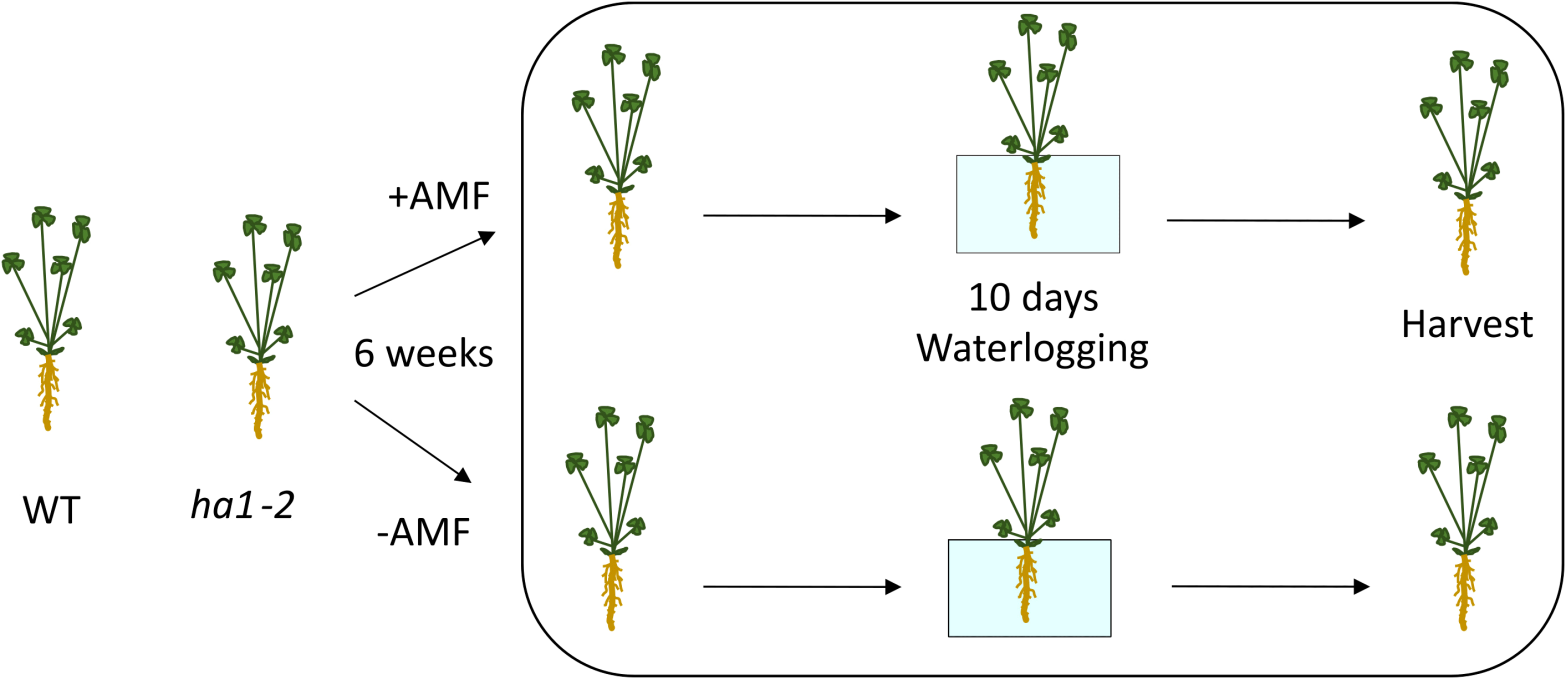
Experimental set up. WT and *ha1-2* mutant lines were cultivated for six weeks with *R. irregularis* fungi. Waterlogging treatment was applied for 10 days using a half-strength Hoagland nutrient solution, maintaining a 1 cm coverage above the soil surface until the harvest day. Control plants were watered regularly. +AMF and -AMF represent treatment with and without *R. irregularis*, respectively.

To explore the influence of *R. irregularis* on *M. truncatula* plants under flooding stress and to understand the interactions between mycorrhizal symbiosis and flooding tolerance in *M. truncatula*, both WT and *ha1-2* mutant lines were grown for six weeks in the presence or absence of *R. irregularis* fungi. Nine seedlings per treatment were transplanted into pots, with three seedlings per pot considered as one biological replicate. Subsequently, the plants underwent a 10-day waterlogging treatment with a half-strength Hoagland nutrient solution covering 1 cm above the soil surface until the harvest day (**Figure 1**; **Supplementary Figure S2**). Since the *ha1-2* mutant is unable to establish a functional symbiosis with mycorrhizal fungi, we opted to avoid a direct comparison between the WT and *ha1-2* mutant due to potential pleiotropic differences between them. The mutants are Tnt1-insertion lines, thus they contain additional Tnt1-insertions apart from the one in the *Mtha-1* gene. To minimize the influence of unintended effects in our analysis, we compared each line to its respective control condition, rather than making direct comparisons between the two genotypes. We believe the current analysis adequately addresses the main research question and provides sufficient comparison for readers to draw meaningful conclusions.

**Figure 2:**
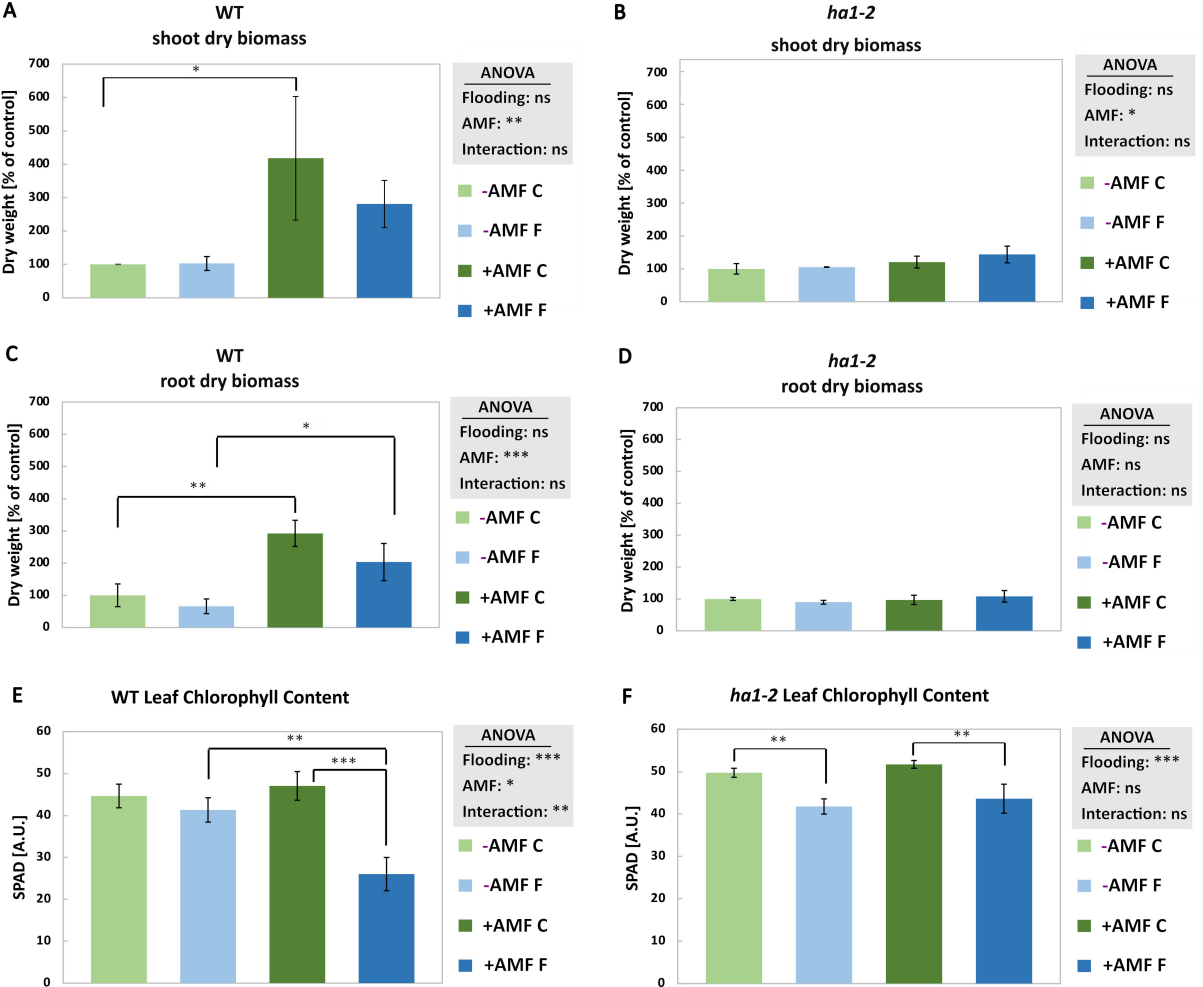
Shoot and root dry biomass and leaf chlorophyll content of WT (**A**, **C** and **E**, respectively) and *ha1-2* (**B**, **D** and **F**, respectively) plants in response to AM fungi and flooding. Plants were harvested after 6 weeks of followed by 10 days of waterlogging. -AMF: without *R. irregularis*, +AMF: with R. irregularis, C: control, F: flooding. The statistical analysis was carried out using 2-way ANOVA. Significance levels are given as ns p>0.05, * p<0.05, ** p<0.01, *** p<0.001. Subsequently, a Tukey’s HSD post hoc test was performed to identify significant differences between all different treatments (multiple comparison), represented as stars on the bars. The error bars represent the standard deviation. Data represent means ± SD (n = 3). Each biological replicate consisted of 3 technical replicates. n.s., not significant.

Under flooding conditions, mycorrhizal plants exhibited increased root and shoot dry biomass when compared with non-mycorrhizal plants subjected to flooding (**Figures 2A, B**). Moreover, *R. irregularis* colonization alone, without functional mycorrhizal Pi transport pathway, did not induce a significant impact on the dry biomass of *ha1-2* mutant plants (**Figures 2C, D**). Furthermore, flooding caused a notable decline in chlorophyll levels compared to non-flooded plants, with a more pronounced decrease observed in mycorrhizal WT plants compared to mycorrhizal *ha1-2* plants (**Figures 2E, F**). Moreover, no significant differences were observed in the root and shoot length of mycorrhizal and non-mycorrhizal plants in response to flooding regardless of the genotype (**Supplementary Figure S3**). These data indicate that AM functional symbiosis, and not necessarily colonization as in the *ha1-2* mutant line, positively affects plant biomass under control conditions but not during flooding. Concerning agricultural applications, this AM-mediated biomass increase might be beneficial for less yield loss under flooding.

### Flooding exhibited no impact on Pi content but repressed the transcript levels of Pi marker gene

**Figure 3:**
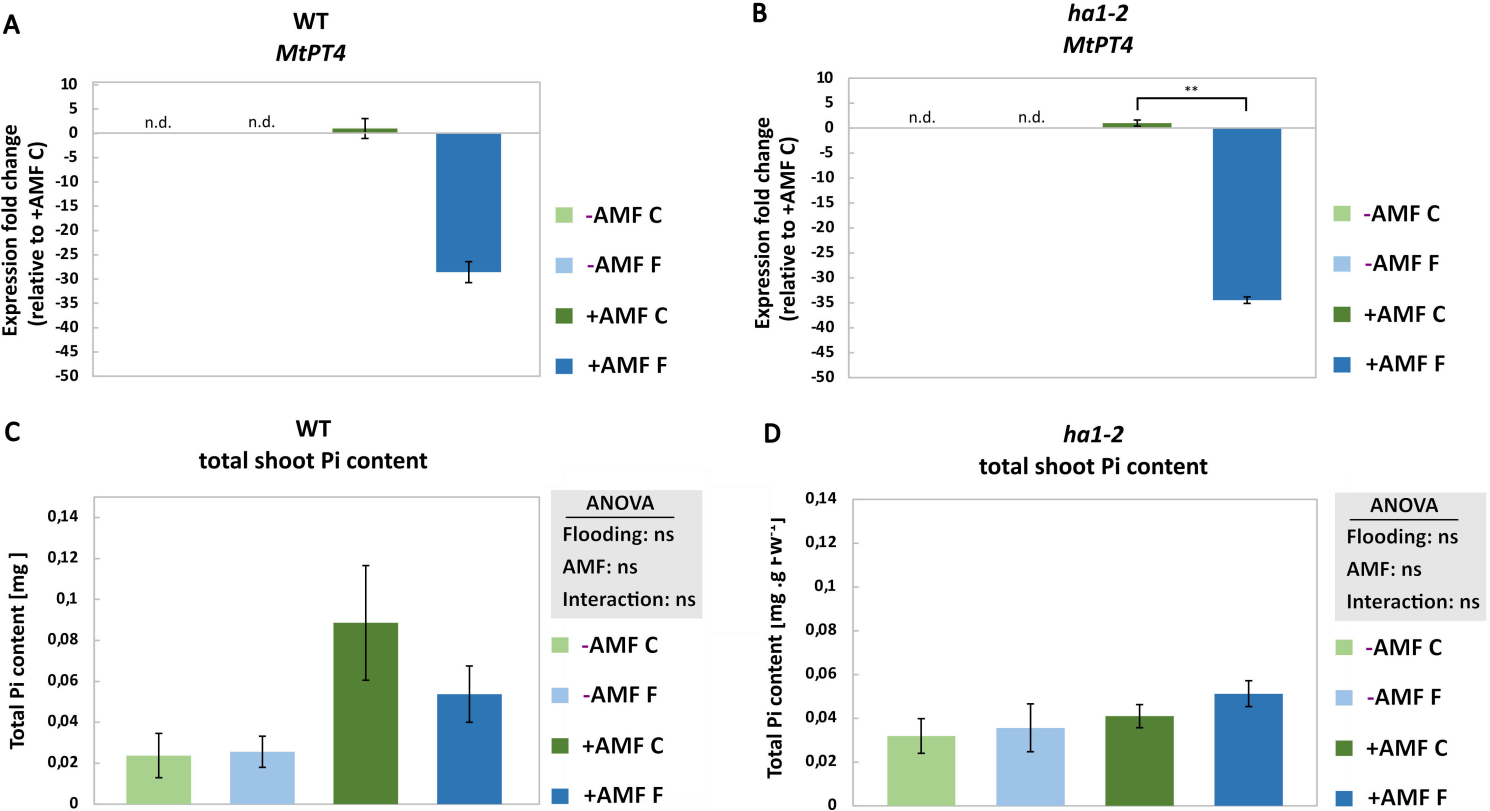
Effect of flooding on the Pi transporter (*MtPT4*) transcript level (**A** and **B**, respectively) and total Pi content (**C** and **D**, respectively) in the leaf of *M. truncatula* in response to AM fungi and flooding. Measurements were conducted on root samples after 6 weeks of growth under phosphate deficiency, both with and without *R. irregularis*, followed by 10 days of waterlogging. -AMF, without mycorrhiza; +AMF, with mycorrhiza; C, control; F, flooding; n.d., not determined. For the measurement of MtPT4 transcripts, normalization was performed using reference genes encoding elongation factor alpha (EF1-α) and glyceraldehyde-3-phosphate dehydrogenase (GAPDH). The control (+AMF C) was normalized to 1 and the statistical analysis was carried out using the student’s t-test, for *MtPT4* transcript level, and two way ANOVA, for total Pi content. Significance levels are given as ns p>0.05, ** p<0.01. Subsequently, a Tukey’s HSD post hoc test was performed to identify significant differences between all different treatments (multiple comparison), represented as stars on the bars. The error bars represent the standard deviation. Data represent means ± SD (n = 3). Each biological replicate consisted of a pool of 3 plants. n.d., not determined.

To evaluate the plant’s Pi status in response to flooding, the transcript level of *MtPT4* involved in mycorrhizal Pi uptake (Javot et al., 2007a) as well as leaf Pi content were measured. Flooding led to the suppression of *MtPT4* transcript levels in both WT, although not statistically significant, and *ha1-2* mutant plants (**Figures 3A, B**). Moreover, neither AM symbiosis nor flooding had any impact on Pi content, irrespective of the genotype (**Figures 3C, D**). This result was expected in the *ha1-2* where no Pi transport is active, nevertheless, the absence of Pi increase in WT plants could potentially be elucidated by considering the prospect of Pi conversion to organic phosphate. It is noteworthy that neither flooding, nor *HA1-2* mutation showed a significant effect on the arbuscule intensity in our study (**Supplementary Figure S4**).

### Flooding exhibited a limited impact on the mycorrhizal structures

**Figure 4:**
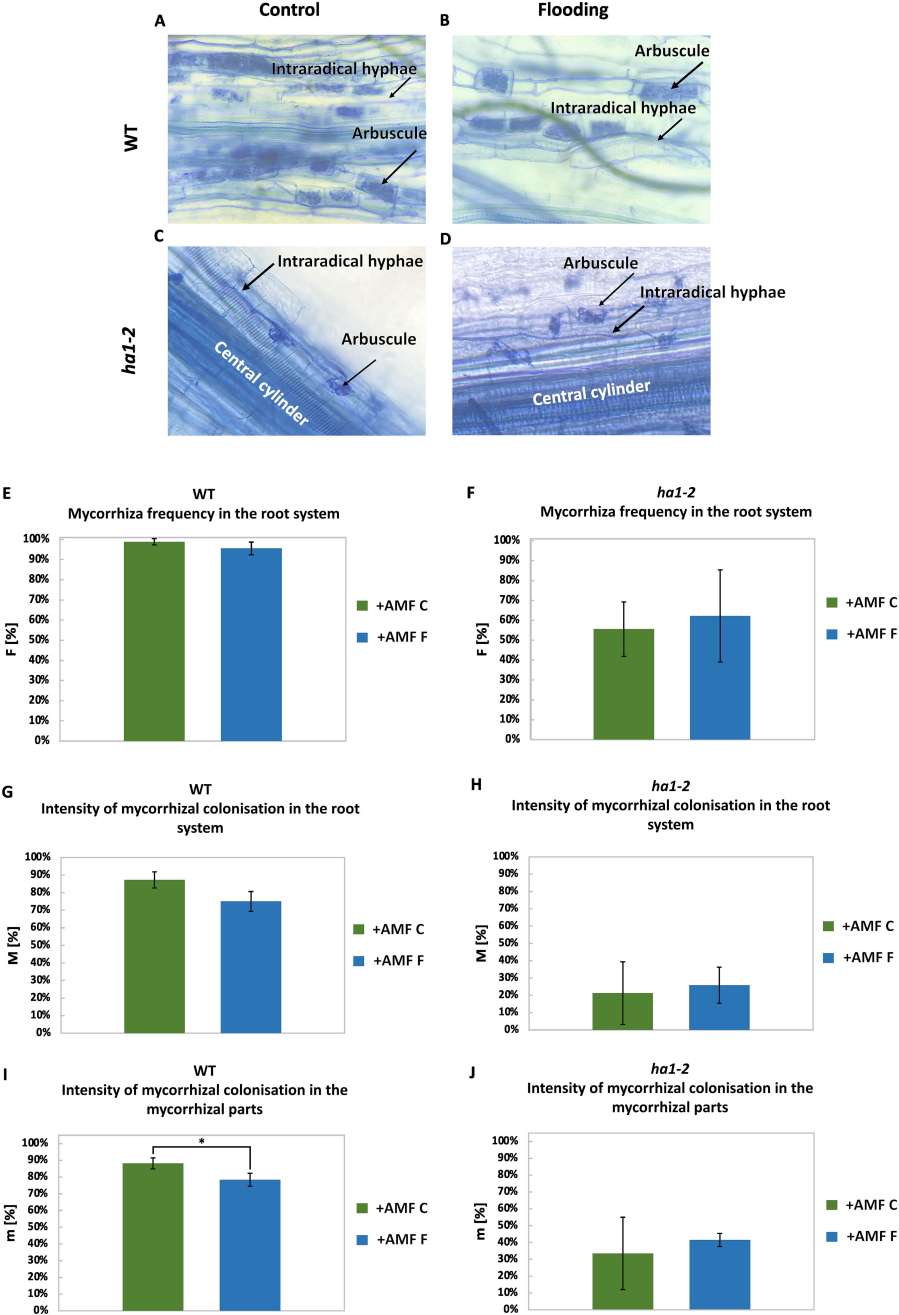
Effects of flooding on the frequency of mycorrhization in the root system (F [%]), intensity of colonization in the root system (M [%]) and in the colonized parts (m [%]) in WT and ha1-2 plants. Differences in the arbuscule structure of the AM fungus R. irregularis in roots of the WT **(A)** and the ha1-2 mutant **(B)** of *M. truncatula* under control and flooding conditions. Arrows indicate one arbuscule per image. Mycorrhizal frequency in the root system **(A)**, intensity of mycorrhizal colonization in the root system **(C)** and in mycorrhizal segments **(E)** of WT and of ha1-2 mutant, respectively **(B**, **D**, **F)**. Moreover, intensity of mycorrhizal colonization in the root system **(G**, **H)** and in the mycorrhizal parts **(I**, **J)** were calculated in WT and *ha1*-2 mutant. Measurements were conducted on root samples after 6 weeks of growth under phosphate deficiency, both with and without *R. irregularis* inoculation, followed by 10 days of waterlogging. +AMF, with R. irregularis; C, control; F, flooding. The statistical analysis was carried out using Student’s t-test (* p<0.05). Error bars represent the standard deviation. Data represent means ± SD. Nine seedlings per treatment were transplanted into pots, with three seedlings per pot considered as one biological replicate.

Next, mycorrhizal structures were evaluated by light microscopy (Trouvelot et al., 1986). WT plants contained fully-developed arbuscules that filled the entire root cell (**Figures 4A, B**). In *ha1-2* plants, however, small, truncated arbuscules were present (**Figures 4C, D**) as described before (Krajinski et al., 2014).

There were no significant differences in the mycorrhizal colonization frequency between flooded and non-flooded plants. It is noteworthy that *ha1-2* mutants exhibited reduced mycorrhization frequency compared to WT (**Figures 4E, F**), due to the absence of a functional mycorrhizal Pi uptake system (Krajinski et al., 2014). Mycorrhization intensity (M) showed no significant difference between flooded and non-flooded plants, however, mycorrhization was notably less intense in *ha1-2* compared to the WT (**Figures 4G, H**).

In the WT plants, a decreased intensity of mycorrhizal colonization in the colonized parts of the root system (m) was observed in flooded roots compared to non-flooded roots, indicating a potential adverse effect of flooding on functional AM symbiosis (**Figure 4I**).

Conversely, in *ha1-2* mutants, there were no significant differences in colonization intensity within the colonized root zones between control and flooded plants (**Figure 4J**), suggesting that the absence of the mycorrhizal Pi uptake system in the *ha1-2* mutants has a greater impact on the mycorrhizal symbiosis than the flooding treatment.

These data suggest that the negative impact of flooding is more influenced by the functional Pi transport from fungi to the plant, as observed in the WT group, rather than colonization alone, as demonstrated in the *ha1-2* line.

### Genes related to nitrogen metabolism and hypoxia response showed modulations in response to AM symbiosis and flooding

**Figure 5:**
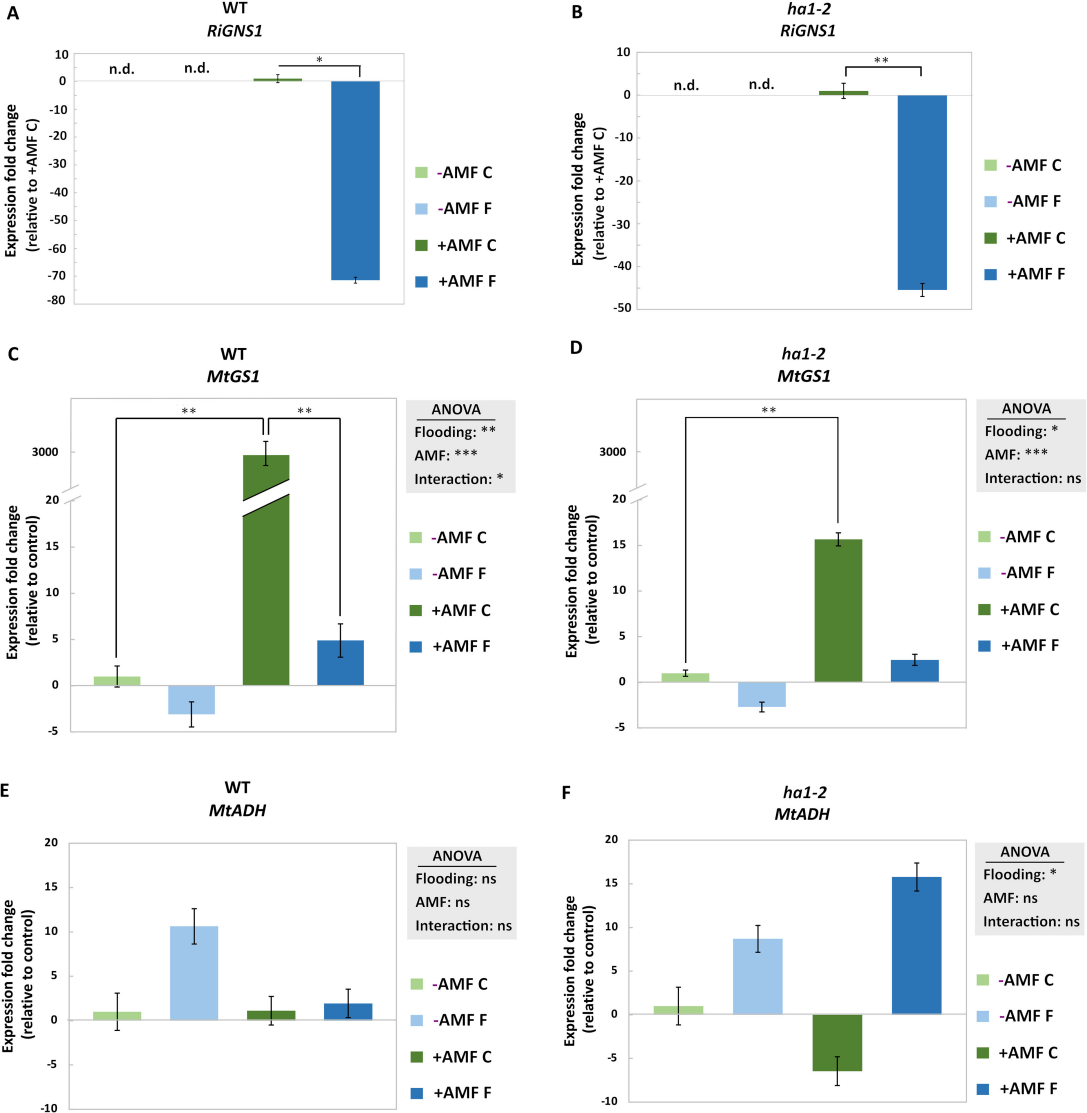
Effect of flooding on the transcript levels of the genes related to nitrogen metabolism and hypoxia response in the roots of mycorrhizal and nonmycorrhizal WT and ha1-2 plants. Transcript levels of glutamine synthetase 1 of the arbuscular mycorrhizal fungus R. irregularis (RiGNS1) in WT and ha1-2 line **(A**, **B)**, the cytosolic glutamine synthetase 1 of M. truncatula (MtGS1) ) in WT and ha1-2 line **(C**, **D)** as well as MtADH in WT and ha1-2 line **(E**, **F)**. Transcript levels were measured in root samples after 6 weeks of growth under phosphate deficiency, both with and without R. irregularis, followed by 10 days of waterlogging. -AMF: without mycorrhiza, +AMF: with mycorrhiza, C: control, F: flooding. n.d.: not determined. The reference genes encoding elongation factor alpha (EF1-α) and glyceraldehyde-3-phosphate dehydrogenase (GAPDH) were used for normalization in the data analysis of transcript levels for M. truncatula genes. Additionally, the transcript level of RirRNA served as the reference for normalizing the R. irregularis marker gene. The control (+AMF C in case of RiGS1 and -AMF C in case of MtGS1 and MtADH1) was normalized to 1 and the statistical analysis was carried out using 2-way ANOVA. Subsequently, a Tukey’s HSD post hoc test was performed to identify significant differences between all different treatments (multiple comparison), represented as stars on the bars. For pairwise comparison (RiGNS1) Student’s t-test was used. Significance levels are given as * p<0.05, ** p<0.01, *** p<0.001. The error bars represent the standard deviation. Data represent means ± SD. Nine seedlings per treatment were transplanted into pots, with three seedlings per pot considered as one biological replicate. nd: not determined, ns: not significant.

Glutamine synthetase 1 (GNS1) is involved in glutamine biosynthesis from ammonium, specifically in the roots (Parniske, 2008). To assess the significance of MtGNS1 in root-specific glutamine biosynthesis and its response to symbiotic interactions we investigated its transcript level in the root of mycorrhizal and non-mycorrhizal plants under control and flooding conditions. Functional symbiosis in WT resulted in a strong upregulation of the *MtGNS1* transcript (~3000-fold) (**Figure 5A**). This upregulation was milder in the *ha1-2* plants (**Figure 5B**). On the other hand, flooding induced a contrary effect, leading to the downregulation of *MtGNS1*, irrespective of AM symbiosis (**Figures 5A, B**). Notably, the *RiGNS1* transcript showed downregulation in response to flooding in both WT and mutant lines, although, this downregulation was more pronounced in the WT plants compared to the *ha1-2* mutant (>4-fold) (**Figures 5C, D**).

We investigated the transcript level of *MtADH1* gene, a hypoxia marker associated with fermentation under low oxygen conditions (Ismond et al., 2003). A higher expression of *MtADH1* was observed under flooding in non-mycorrhizal WT plants when compared to mycorrhizal-flooded plants, however, these changes were not statistically significant (**Figures 5E, F**). Additionally, an elevated expression of *MtADH1* transcripts was noted in the *ha1-2* mutant under flooding compared to the flooded WT, although statistical significance was not observed (**Figure 5F**) which might suggest a potential repression of the hypoxia response by functional symbiosis.

## Discussion

This study provides insights into the complex interactions between AM fungi symbiosis, flooding stress, and plant responses. Flooding stress poses significant challenges to plants by restricting oxygen availability, altering nutrient uptake, and disrupting physiological processes (Safavi-Rizi et al., 2024). Moreover, flooding can affect the establishment and maintenance of AM symbiosis, which is formed by the majority of terrestrial plants and leads to improved nutrition and increased tolerance to various stresses (Wang et al., 2021). Some studies have reported reduced mycorrhizal colonization under flooding conditions, possibly due to limitations in oxygen diffusion and altered root exudation patterns (Vallino et al., 2014; Wang et al., 2021). Moreover, flooding stress can affect nutrient availability and uptake, which in turn, may impact mycorrhizal associations (Aslam et al., 2023). Here we aim to investigate the nuanced responses of mycorrhizal plants to flooding stress using physiological and molecular parameters related to growth promotion and stress susceptibility. To distinguish between the effects of flooding stress and the effects of enhanced Pi uptake facilitated by mycorrhizal symbiosis, we incorporated an *M. truncatula* mutant, *ha1-2*, into the study. This mutant is incapable of phosphate uptake via the mycorrhizal pathway (Krajinski et al., 2014; Wang et al., 2014) resulting in reduced root colonization while still permitting colonization *per se*.

The present study focuses on the effects of AM symbiosis on the hypoxia response of *M. truncatula*. WT and the *ha1-2* mutant plant lines were cultivated with *R. irregularis* fungi for six weeks. Subsequently, both sets of plants underwent a 10-day waterlogging treatment, using Hoagland nutrient solution covering up to 1 cm above the soil surface. We evaluated several phenotypic, physiological, and molecular parameters to investigate the effect of *R. irregularis* on flooding response of *M. truncatula* WT and *ha1-2* mutant line.

### Genotype dependent impacts of AM fungi symbiosis and flooding on growth and physiological response of *M. truncatula*

While mycorrhizal WT plants exhibited higher biomass compared to non-mycorrhizal plants under flooding, the reduction in biomass was more pronounced in mycorrhizal plants (**Figures 2A, C**). This suggests that, despite the potential benefits of AM-mediated biomass increase, such symbiosis does not improve growth under flooding conditions. *M. truncatula* WT plants, which are able to take up phosphate via arbuscular mycorrhizal pathway, showed an increase in dry biomass compared to respective control plants (**Figures 2A, C**). In the *ha1-2* mutant, this positive effect on growth due to the AM symbiosis was not observed. In fact, the biomass of the mycorrhizal *ha1-2* plants resembled that of non-mycorrhizal plants (**Figures 2B, D**). Our results suggest increased Pi provided by the mycorrhizal uptake pathway leads to a higher biomass under control but not under flooding conditions (**Figures 2A, C**). This trend aligns with previous studies on the influence of mycorrhizal symbioses on flooding response (Krajinski et al., 2014). Research on rice has indicated a detrimental impact of mycorrhizal colonization under hypoxia, resulting from a reduction in Pi utilization by rice plants (Wang et al., 2021). Hence, while higher biomass doesn’t necessarily enhance plant survival during flooding, it is contingent upon other factors such as Pi availability to the plants.

In both WT and the *ha1-2* mutant, flooding led to a reduction in chlorophyll content compared to the unflooded control (**Figures 2E, F**). The negative effect of oxygen deprivation in the root zone on the chlorophyll content was also shown in studies on *M. sativa* varieties in which the plants showed a reduction in chlorophyll content of approximately 20% after 9 to 11 days of waterlogging (Smethurst and Shabala, 2003). This negative effect of hypoxia can be explained, to some extent, by the impairment of root functionality in nutrient uptake and transport into the leaves due to insufficient internal aeration of the roots (Striker and Colmer, 2016). Moreover, accumulation of reactive oxygen species in the leaves under oxygen deprivation can lead to leaf senescence and chlorophyll degradation (Loreti et al., 2016). We observed that WT mycorrhizal plants showed a more drastic chlorophyll reduction under flooding compared to the non-mycorrhizal flooded plants (**Figure 2E**). This pattern can probably be due to the competition between the AM fungi and plant for the carbon derived from photosynthesis as well as limited Pi supply from the AM fungi to the plant under flooding (Tinker, 1975; Wang et al., 2021). Flooding leads to disruption of photosynthesis and C level which can eventually affect mycorrhiza performance in paddy fields due to lower C level and a higher C competition between plant and AMF, similar to shading condition (Korhonen et al., 2004; Konvalinková et al., 2015). Moreover, alterations in soil pH triggered by flooding may lead to increased mobility of nutrients such as Pi as well as microbial activities which potentially reduce the dependence of plants on the mycorrhizal mediated phosphorus uptake (Minasny et al., 2016; Ding et al., 2019). It is shown that flooding negatively affects the spread of extraradical hyphae of AM fungi probably due to lower oxygen concentration in the flooded soil which can further contribute to the reduced C and P exchange between plant and AM fungi (Vallino et al., 2014).

### Energy-consuming processes and hypoxia response gene showed downregulation under hypoxia

To explore the impact of flooding on Pi transport, we investigated the expression levels of *MtPT4*, a pivotal gene associated with Pi transport in arbuscule containing cells. We observed a notable downregulation of *MtPT4* in both mycorrhizal WT and *ha1-2* mutant plants under flooding compared to control conditions (**Figures 3C, D**). This downregulation indicates a potential modulation of Pi uptake pathway in mycorrhizal plants in response to flooding stress. The downregulation of Pi transporter gene *MtPT4* under flooding suggests a coordinated reprogramming of nutrient uptake pathways. This could be a direct response to the hypoxic environment, in which the plant limits energy-consuming processes, such as Pi uptake, to conserve resources.

In addition, the expression of the gene encoding cytosolic glutamine synthetase *(MtGS1)* was analyzed to explore the effect of mycorrhizal colonization and flooding. The data showed a higher expression in WT and *ha1-2* with *R. irregularis* under control conditions compared to the control non-mycorrhizal plants (**Figure 5**). This suggests that more NH_4_^+^ could be taken up under aerobic conditions in the presence of AM fungi (Bücking and Kafle, 2015). In WT, this positive effect was much stronger than in *ha1-2*, suggesting a link between NH_4_^+^ and Pi transport (Javot et al., 2007b). In symbiosis, N and Pi transfer shows remarkable similarities. Both elements are absorbed by extraradical hyphae, then transported to intraradical hyphae, stored as poly-P or arginine. Finally, ionic forms (Pi or NH_4_^+^) are released to the plant apoplast and taken up by specialized transporters (Javot et al., 2007b). Moreover, it is shown that the upregulation of the fungal Pi-transporter relies on the presence of N (Olsson et al., 2005).

However, under hypoxia, *MtGS1* exhibited downregulation during flooding compared to the control conditions across all treatments. Remarkably, mycorrhizal treatments during flooding showed comparable gene expressions to non-mycorrhizal treatments under the same conditions, observed in both WT and *ha1-2* plants (**Figure 5**). It is postulated that, during the plant’s late hypoxia response, ATP-consuming activities are curtailed to conserve energy, with ammonium assimilation through GS being one of these energy-intensive processes. The downregulation of *MtGS1* under flooding reflects the plant’s strategy of conserving energy under hypoxic stress. This suggests a shift in metabolic priorities, where carbon and nitrogen assimilation are temporarily reduced to minimize ATP consumption. In this context, the AM symbiosis may modulate these responses by influencing both nutrient availability and carbon allocation.

Hypoxia enhances *ADH* expression and alcoholic fermentation, ensuring the maintenance of energy metabolism within the cell (Diab and Cukier, 2013; Loreti and Perata, 2020). Mutants in maize, rice, and Arabidopsis with impaired ADH and PDC function exhibit rapid death under oxygen deprivation, underscoring the critical role of ethanolic fermentation in flooding tolerance (Fukao and Bailey-Serres, 2004). In this study, up-regulation of *MtADH* was observed under flooding compared to aerobic conditions in both WT and *ha1-2* mutants, except in the mycorrhizal WT plants (**Figure 5**). This might hint that a functional AM symbiosis, involving Pi transport, suppresses the plant hypoxia response. It is suggested that the allocation of carbon to the symbiosis may lead to a reduction in the available carbon for sustaining glycolysis. As a result, there is a diminished supply of pyruvate as the initial substrate for alcoholic fermentation, and consequently, there is no requirement for an increase in ADH activity (Fukao and Bailey-Serres, 2004). Prior investigations have documented a reduction in carbon allocation from host plants to AM fungi during flooding conditions (Bao et al., 2019). Our data suggests that functional arbuscular mycorrhizal symbiosis suppresses plant hypoxia response by altering carbon allocation and probably reduces the need for an increased *ADH* expression under flooding conditions.

Chickpea seedlings inoculated with mycorrhizae have demonstrated enhanced tolerance to flooding compared to non-inoculated controls. These seedlings exhibited lower root ethanol accumulation, a more gradual decline in overall plant health, and greater retention of viable root mass. In non-wetland species, ethanol buildup is associated with flooding-induced stress; however, the actual damage is likely caused by acetaldehyde, a toxic intermediate (Crawford and Zochowski, 1984; Perata and Alpi, 1993). Mycorrhizal colonization has been shown to mitigate ethanol accumulation in other plant species, such as peach seedlings, with similar findings reported in *Casuarina equisetifolia* (Osundina, 1997; Rutto et al., 2002).

Research into the effects of flooding on the root characteristics of mycorrhizal versus non-mycorrhizal tomato plants has shown that mycorrhizal plants adapt more effectively to flooding. This adaptation may be attributed to modifications in root morphology and physiological responses (Calvo-Polanco et al., 2014). Although this study did not examine aquaporin expression or root hydraulic conductivity, plant hormones such as ethylene and indole-3-acetic acid (IAA) likely play key roles in regulating root function under hypoxic conditions. These results highlight the potential of mycorrhizal inoculation to improve flood tolerance; however, further research is needed to clarify the underlying mechanisms.

## Conclusion

Our study investigated the role of AM colonization in *M. truncatula* responses to flooding, uncovering key interactions between mycorrhizal symbiosis and flooding tolerance. Under normal conditions, AM symbiosis significantly enhanced plant biomass production, with mycorrhizal wild-type plants exhibiting increased dry biomass compared to non-mycorrhizal controls. However, during flooding stress, these benefits were lost, and mycorrhizal plants experienced a more pronounced decline in biomass. This suggests that while AM symbiosis supports growth under optimal conditions, it may exacerbate stress effects during flooding. The reduced plant growth under flooding conditions may stem from altered soil pH, disrupted microbial activity affecting nutrient availability, particularly phosphate, and shifts in carbon allocation between the plant and fungus. Additionally, the upregulation of nitrogen transport and hypoxia-related genes, such as MtGNS1, in mycorrhizal plants highlights the complex role of AM symbiosis in modulating stress responses. Our findings also suggest that AM symbiosis may indirectly benefit plants by improving vigor prior to flooding, potentially aiding survival during initial stress. Future studies should explore how the intensity and duration of flooding influence AM symbiosis, focusing on nutrient dynamics, hypoxia adaptation, and metabolic adjustments. Investigating specific AM fungal strains could reveal tailored strategies for enhancing flooding tolerance in *M. truncatula*. Moreover, molecular studies on AM-induced changes in root architecture, nutrient cycling, and stress hormone signaling will clarify how mycorrhizal fungi impact plant resilience. In agricultural contexts, optimizing AM symbiosis may provide sustainable strategies to improve crop productivity and reduce fertilizer dependency in flood-prone regions, promoting resilience in the face of climate change.

## Data Availability

The raw data supporting the conclusions of this article will be made available by the authors, without undue reservation.
