# Supplementary material for: The impact of arbuscular mycorrhizal colonization on flooding response of Medicago truncatula
Source: Front Plant Sci. 2025 Jan 8;15:1512350. doi: 10.3389/fpls.2024.1512350 (PMC11750877; doi:10.3389/fpls.2024.1512350)
Supplement: Supplementary file 1 [file Table1.docx]

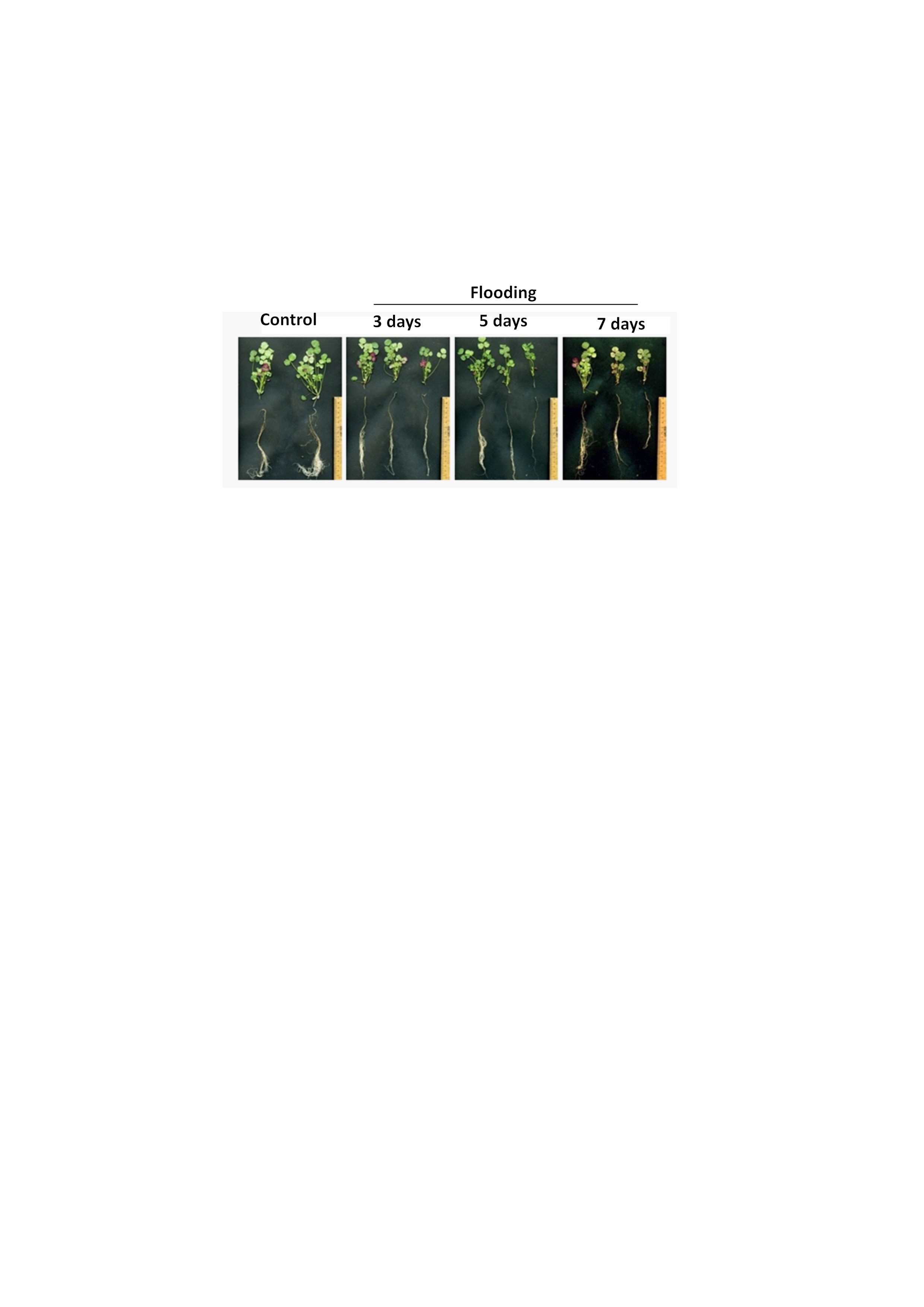


**Supplementary Figure S1.** **Impact of Flooding duration on the phenotype of *M. truncatula***: Wild-type (WT) Medicago truncatula plants were cultivated for 19 days without arbuscular mycorrhizal (AM) fungi. Following this growth period, the plants' roots were subjected to waterlogging (flooding) for varying durations of 3, 5, and 7 days.
