# Supplementary material for: The impact of arbuscular mycorrhizal colonization on flooding response of Medicago truncatula
Source: Front Plant Sci. 2025 Jan 8;15:1512350. doi: 10.3389/fpls.2024.1512350 (PMC11750877; doi:10.3389/fpls.2024.1512350)
Supplement: Supplementary file 2 [file Table2.docx]

**
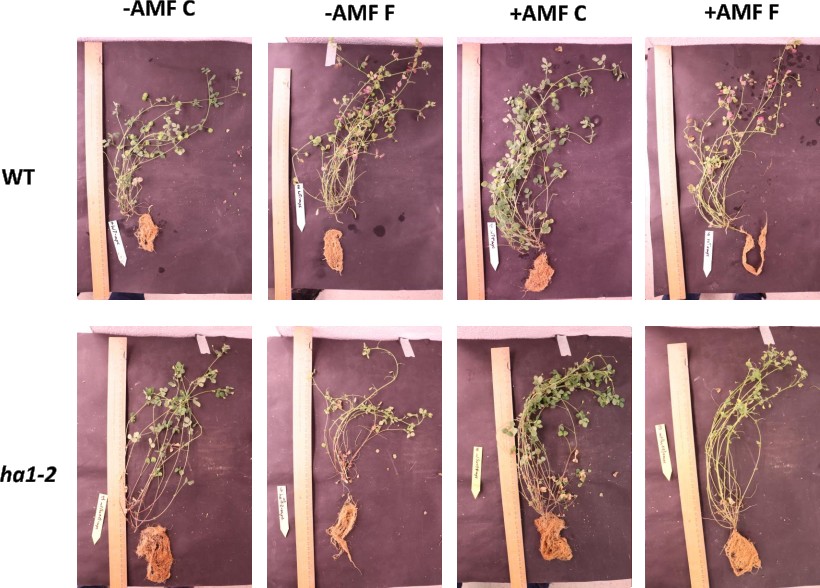
**

**Supplementary Figure S2. Phenotype of *M. truncatula* plants, WT and *ha1-2* line, in response to AM fungi and flooding.** Plants were grown for 6 weeks in the with or without AM fungi followed by 10 days of waterlogging. -AMF: without *R. irregularis,* +AMF: with *R. irregularis*, C: control, F: flooding. It is noteworthy that the phenotype of the plants are obtained from a parallel experiment conducted under the exact same condition described in this study. The root samples were folded after washing and therefore their size on the photo does not represent their length.
