# Supplementary material for: The impact of arbuscular mycorrhizal colonization on flooding response of Medicago truncatula
Source: Front Plant Sci. 2025 Jan 8;15:1512350. doi: 10.3389/fpls.2024.1512350 (PMC11750877; doi:10.3389/fpls.2024.1512350)
Supplement: Supplementary file 3 [file Table3.docx]

**
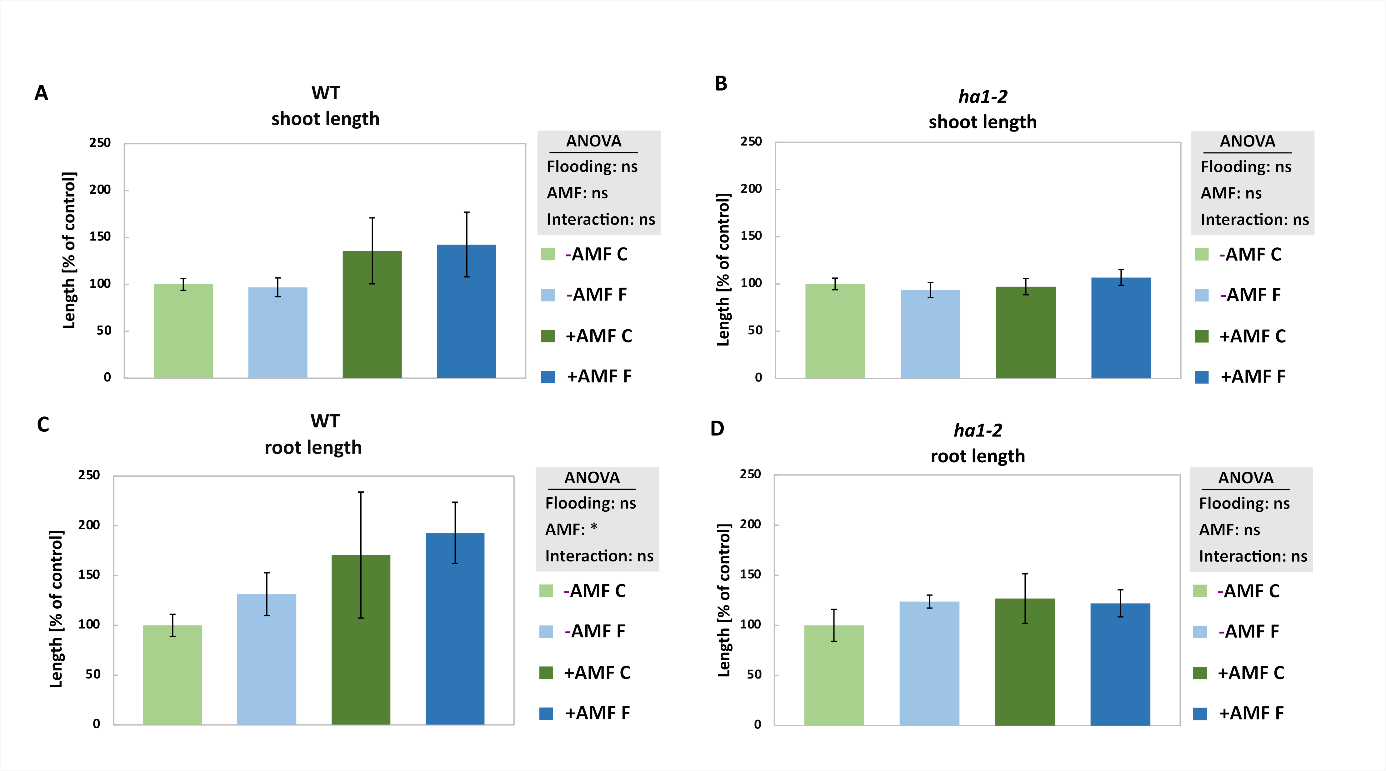
**

**Supplementary Figure S3. Effects of flooding on the shoot and root length in WT and *ha1-2* plants.** Measurements were conducted on shoot and root samples after 6 weeks of growth under phosphate deficiency, both with and without *R. irregularis*, followed by 10 days of waterlogging. -AMF: without mycorrhiza, +AMF: with mycorrhiza, C: control, F: flooding. n.d.: not determined. The control was normalized to 1 and the statistical analysis was carried out using 2-way ANOVA. Significance levels are given as ns p>0.05, * p<0.05, ** p<0.01, *** p<0.001 and **** p<0.0001. The error bars represent the standard deviation. Data represent means ± SD (n = 3). Each biological replicate consisted of a pool of 3 technical replicates.

| **Primer name** | **Sequence (5' to 3')** | **Reference** |
| --- | --- | --- |
| **Hypoxia markers** |  |  |
| **MtADH_F** | GGGACTATGTTCTCAATCTGG | Berger et al., 2020a |
| **MtADH_R** | TAGGTACCAAATGTCACAGTCTC |  |
| **MtPDC_F** | GCCCCGCGTTAAGATCAAC |  |
| **MtPDC_R** | CCAAGTTATTCACCACTGCCT |  |
| **Phytoglobins** |  |  |
| **MtPGB1.2_F** | GGACAATGCCAATTTGATAAGCAG |  |
| **MtPGB1.2_R** | CTGGTGGAGCAATCTCAAGG |  |
| **MtPGB1.3_F** | TTCTCATGACATGTGAATCAGC |  |
| **MtPGB1.3_R** | GTGACCACATTTCAGGTAATGC |  |
| **MtPGB3.1_F** | GCTTCATCACACACACATAC |  |
| **MtPGB3.1_R** | AATCATGATCTATATCTGAAATGTT |  |
| **MtPGB3.2_F** | AACTTTATAAGTTTTCTTTTGTTTG |  |
| **MtPGB3.2_R** | GATAGACATATAGACGTTCAATCTT |  |
| **Nitrogen metabolism** |  |  |
| **MtNR1_F** | GTTCAGTTTGCAGTAAAGCC | Berger et al., 2020b |
| **MtNR1_R** | ATACATACAGCGTCGTACTC |  |
| **MtNR2_F** | CCACCTATGATTCAATTTGCTG |  |
| **MtNR2_R** | TCTATTACTTGCCCTAGAACAC |  |
| **MtNR3_F** | GCATGGGATCTGGCTAATAACAC |  |
| **Mt_NR3_R** | TTATTCTTAGGGTCTGGGTCAGAG |  |
| **MtGS1_F** | CTTGACCTCTCCGAAACCA |  |
| **MtGS1_F** | CTTGGGAAGCTGTGAAGGG |  |
| **Phosphate transporter** |  |  |
| **MtPt4_F** | CAAGAAAGATTAGACGCGCAA | This study |
| **MtPt4_R** | GTTTCCGTCACCAAGAAC-GTG |  |
| **H+-ATPase** |  |  |
| **MtHA1-F** | CTTTGTGCTTTTCGCACATAACAT |  |
| **MtHA1_R** | AGACAAAAAAATATAAAACAATAGCCAATG |  |
| **Housekeeping genes** |  |  |
| **MtGAPDH_F** | TGCCTACCGTCGATGTTTCAGT |  |
| **MtGAPDH_R** | TTGCCCTCTGATTCCTCCTTG |  |
| **MtEF-1 alpha** | GACAAGCGTGTGATCGAGAGATT |  |
| **MtEF-1 alpha** | TTTCACGCTCAGCCTTAAGCT |  |
| ***Rizophagus irregularis* markers** |  |  |
| **RiGNS1-F** | AACGGTTAACTGGTAGACAC |  |
| **RiGNS1-R** | TAATTTGAGTGACACGGTAAGG |  |
| **Ri rRNA-F** | GTATGCCTGTTTGAGGGTCAGTATT |  |
| **Ri rRNA-R** | AAACTCCGGAACGTCACTAAAGAG |  |

**Supplementary Table 1. List of all primers used.**
