# Supplementary material for: The impact of arbuscular mycorrhizal colonization on flooding response of Medicago truncatula
Source: Front Plant Sci. 2025 Jan 8;15:1512350. doi: 10.3389/fpls.2024.1512350 (PMC11750877; doi:10.3389/fpls.2024.1512350)
Supplement: Supplementary file 4 [file Table4.docx]

**
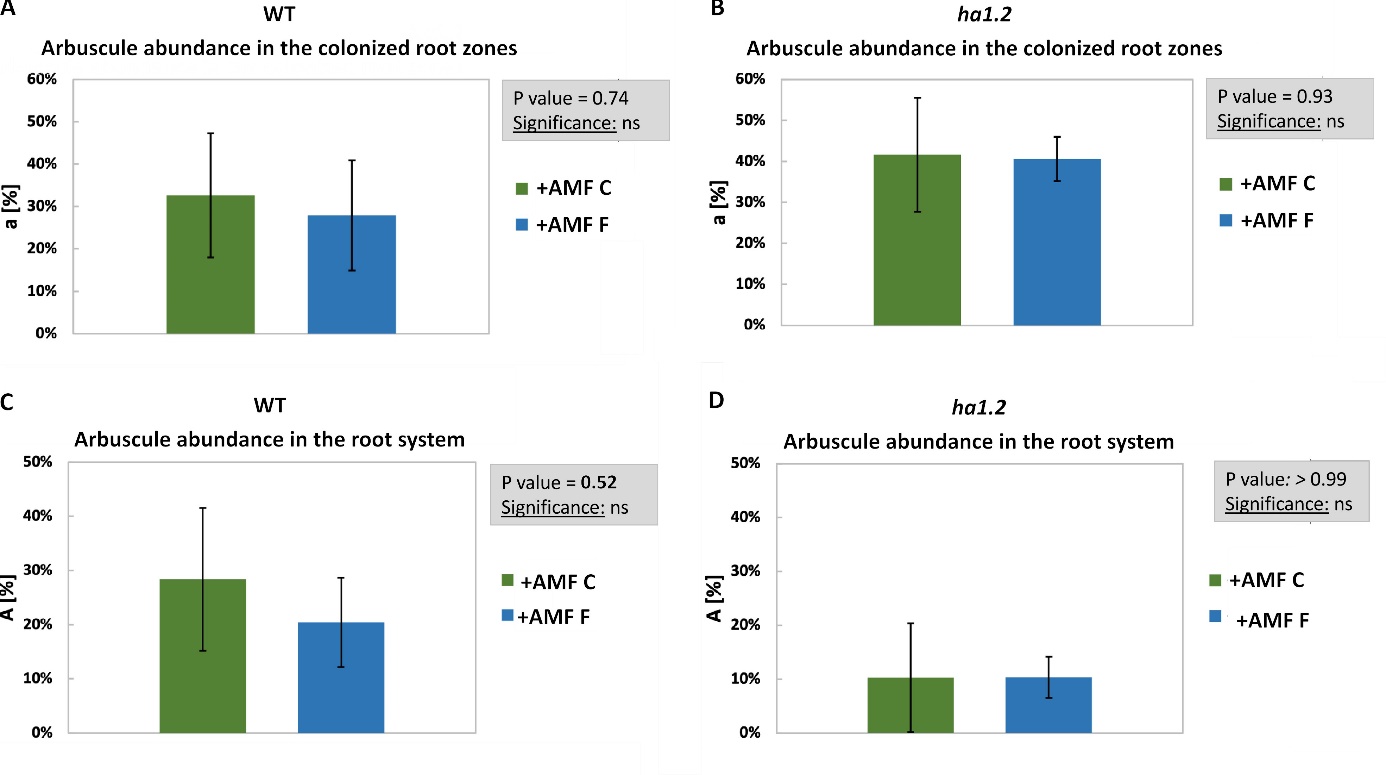
**

**Supplementary Figure S4**. **The effect of flooding on the arbuscule abundance in *M. truncatula* roots**. Arbuscule abundance in the colonized root zones (a%) of WT **(A)** and *ha1-2* line **(B)**. Arbuscule abundance in the root system (A%) of WT **(C)** and *ha1-2* line **(D)**. Arbuscular abundance were calculated using the Trouvelot et al. (1986) method [22], as described in materials and methods section. Measurements were conducted on root samples after 6 weeks of growth under phosphate deficiency, followed by 10 days of waterlogging. +AMF: with *R. irregularis*, C: control, F: flooding. The statistical analysis was carried out using Student’s t-test (* p<0.05, ** p<0.01, *** p<0.001 and **** p<0.0001). Error bars represent the standard deviation. Data represent means ± SD (n = 3). Each biological replicate consisted of a pool of 3 plants.
