# Supplementary material for: The impact of arbuscular mycorrhizal colonization on flooding response of Medicago truncatula
Source: Front Plant Sci. 2025 Jan 8;15:1512350. doi: 10.3389/fpls.2024.1512350 (PMC11750877; doi:10.3389/fpls.2024.1512350)
Supplement: Supplementary file 5 [file Table5.docx]

| **Primer name** | **Sequence (5' to 3')** | **Reference** |
| --- | --- | --- |
| **Hypoxia markers** |  |  |
| **MtADH_F** | GGGACTATGTTCTCAATCTGG | [45] |
| **MtADH_R** | TAGGTACCAAATGTCACAGTCTC |  |
| **MtPDC_F** | GCCCCGCGTTAAGATCAAC | [45] |
| **MtPDC_R** | CCAAGTTATTCACCACTGCCT |  |
| **Nitrogen metabolism** |  |  |
| **MtGS1_F** | CTTGACCTCTCCGAAACCA | [46] |
| **MtGS1_F** | CTTGGGAAGCTGTGAAGGG |  |
| **Phosphate transporter** |  |  |
| **MtPt4_F** | CAAGAAAGATTAGACGCGCAA | [10]* |
| **MtPt4_R** | GTTTCCGTCACCAAGAAC-GTG |  |
| **H+-ATPase** |  |  |
| **MtHA1-F** | CTTTGTGCTTTTCGCACATAACAT | [45] |
| **MtHA1_R** | AGACAAAAAAATATAAAACAATAGCCAATG |  |
| **Housekeeping genes** |  |  |
| **MtGAPDH_F** | TGCCTACCGTCGATGTTTCAGT | [10]* |
| **MtGAPDH_R** | TTGCCCTCTGATTCCTCCTTG |  |
| **MtEF-1 alpha** | GACAAGCGTGTGATCGAGAGATT | [10]* |
| **MtEF-1 alpha** | TTTCACGCTCAGCCTTAAGCT |  |
| ***Rizophagus irregularis* markers** |  |  |
| **RiGNS1-F** | AACGGTTAACTGGTAGACAC | [47] |
| **RiGNS1-R** | TAATTTGAGTGACACGGTAAGG |  |
| **RirRNA-F** | GTATGCCTGTTTGAGGGTCAGTATT | [10]* |
| **RirRNA-R** | AAACTCCGGAACGTCACTAAAGAG |  |

**Supplementary Table 1. List of all primers used.**

**References**

45. Berger A, Guinand S, Boscari A, Puppo A, Brouquisse R. Medicago truncatula Phytoglobin 1.1 controls symbiotic nodulation and nitrogen fixation via the regulation of nitric oxide concentration. New Phytol. 2020;227(1):84-98.

46. Berger A, Boscari A, Horta Araújo N, Maucourt M, Hanchi M, Bernillon S, Rolin D, Puppo A, Brouquisse R. Plant Nitrate Reductases Regulate Nitric Oxide Production and Nitrogen-Fixing Metabolism During the Medicago truncatula-Sinorhizobium meliloti Symbiosis. Front Plant Sci. 2020;11:1313.

47. Tian C, Kasiborski B, Koul R, Lammers PJ, Bücking H, Shachar-Hill Y. Regulation of the nitrogen transfer pathway in the arbuscular mycorrhizal symbiosis: gene characterization and the coordination of expression with nitrogen flux. Plant Physiol. 2010;153(3):1175-87.

*: available in the manuscript reference list.
